# Epithelial uptake leads to fungal killing *in vivo* and is aberrant in COPD-derived epithelial cells

**DOI:** 10.1016/j.isci.2024.109939

**Published:** 2024-05-16

**Authors:** Margherita Bertuzzi, Gareth J. Howell, Darren D. Thomson, Rachael Fortune-Grant, Anna Möslinger, Patrick Dancer, Norman Van Rhijn, Natasha Motsi, Alice Codling, Elaine M. Bignell

**Affiliations:** 1 Manchester Fungal Infection Group, Faculty of Biology, Medicine and Health, The University of Manchester, Core Technology Facility, Grafton Street, Manchester M13 9NT, UK; 2 Flow Cytometry Core Facility, Faculty of Biology, Medicine and Health, Core Technology Facility, Grafton Street, Manchester M13 9NT, UK

**Keywords:** Respiratory medicine, Mycology, Cell biology

## Abstract

Hundreds of spores of *Aspergillus fumigatus (Af)* are inhaled daily by human beings, representing a constant, possibly fatal, threat to respiratory health. The small size of *Af* spores suggests that interactions with alveolar epithelial cells (AECs) are frequent; thus, we hypothesized that spore uptake by AECs is important for driving fungal killing and susceptibility to *Aspergillus*-related disease. Using single-cell approaches to measure spore uptake and its outcomes *in vivo*, we demonstrate that *Af* spores are internalized and killed by AECs during whole-animal infection. Moreover, comparative analysis of primary human AECs from healthy and chronic obstructive pulmonary disease (COPD) donors revealed significant alterations in the uptake and killing of spores in COPD-derived AECs. We conclude that AECs contribute to the killing of *Af* spores and that dysregulation of curative AEC responses in COPD may represent a driver of *Aspergillus*-related diseases.

## Highlights

• Alveolar epithelial cells (AECs) internalize and kill *A. fumigatus* in mice
• COPD significantly impacts uptake and killing of *A. fumigatus* by primary human AECs
• AECs dysregulation in COPD may represent a driver for *A. fumigatus* persistence

## Graphical abstract

**Figure:**
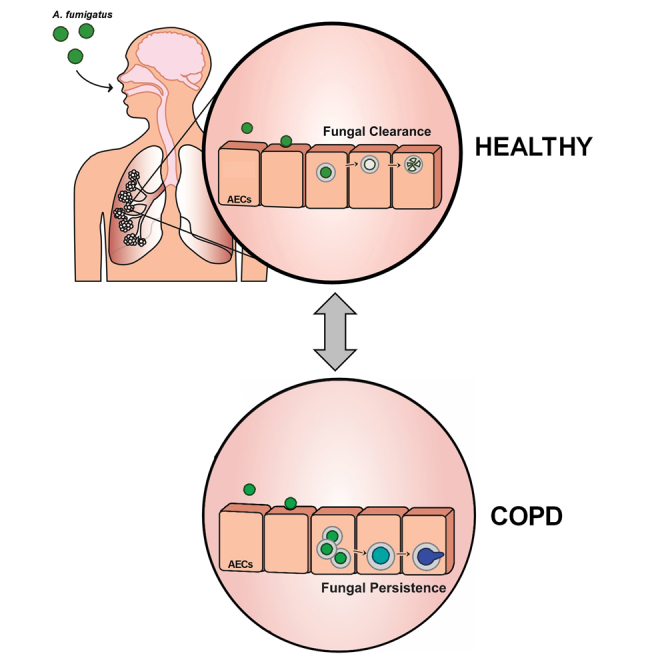

## Introduction

The mold pathogen *Aspergillus fumigatus* (*Af*) causes a broad range of human diseases, whose individual manifestations and mortalities are governed by the host immune status. Invasive aspergillosis (IA) is associated with the highest mortality rates and causes an estimated 300,000 deaths per annum,^1^^,^^2^ of which 10% occur in patients with acute leukemia and recipients of solid organ^3^^,^^4^^,^^5^ or allogenic hematopoietic stem cell transplants and 3.9% occur in patients with chronic obstructive pulmonary disease (COPD).^6^^,^^7^^,^^8^ Allergic bronchopulmonary aspergillosis (ABPA) affects more than 4 million asthmatic and cystic fibrosis sufferers.^9^^,^^10^ Chronic pulmonary aspergillosis (CPA) exacerbates pre-existing structural and immunological lung defects, and it is estimated to affect more than 3 million people worldwide, with up to 50% mortality in the first 5 years after diagnosis.^11^^,^^12^

All disease manifestations are unified by a common pattern of infection, which initiates with the inhalation of fungal conidia and eventually leads to the destruction of the pulmonary parenchyma. *Af* conidia are abundant in the airborne microflora^13^ and have the ideal diameter (2–3 μm) for deep deposition within the alveoli.^14^^,^^15^ In healthy individuals, inhaled conidia are efficiently cleared by the concerted action of the innate immune system, mainly by macrophages and neutrophils.^16^^,^^17^^,^^18^ However, alveolar macrophages (AMs) only constitute ∼3.25% of the total cell number in the alveoli^19^ and are therefore unlikely to be the first cell type encountered by inhaled spores. Macrophage ablation in mice using clodronate^17^ and *in silico* models of infection^20^^,^^21^ indicate that AMs likely migrate too slowly and randomly in the absence of a chemoattractant signal to fully protect against *Af* infection. Furthermore, *in silico*^21^ and murine studies^17^^,^^22^ indicate that recruitment of neutrophils to the site of infection occurs downstream of preceding inflammatory signals. Conversely, the contact of alveolar epithelial cells (AECs) with inhaled *Af* conidia is immediate, extensive, and likely prolonged. Recent *in silico* models^20^^,^^23^^,^^24^ and *in vitro* studies^25^^,^^26^ suggest that AECs play a key role in initiating fungal clearance and host responses by releasing cytokines for immune cell recruitment during infection.

We and others reported that *in vitro* cultured *Af* spores are internalized by the A549 alveolar type-II like cell line^27^^,^^28^^,^^29^^,^^30^^,^^31^ and by 16HBE14o-transformed human bronchial epithelial cells,^32^^,^^33^ both of which internalize ∼30%–50% of the spores they come into contact with. Population-scale analyses of *Af*-AECs interactions *in vitro* indicate that the majority of the internalized spores are killed, but a small proportion (3%) survives and germinates inside acidic organelles,^31^ thereby suggesting that AECs might serve as a fungal reservoir for latent occupation and immune evasion.^34^^,^^35^^,^^36^ It was previously reported that bronchial epithelial cells carrying a non-functional mutated version of the cystic fibrosis transmembrane conductance regulator (CFTR, D508F) are impaired in the uptake and killing of internalized conidia, thereby strongly supporting a curative role for epithelial activities.^37^ Recent research into diverse respiratory pathogens has demonstrated that the respiratory epithelium orchestrates a multifaceted and active response to the presence of inhaled pathogens and highlighted that non-professional phagocytic activities of airway epithelial cells differentially impact infection outcomes in multiple disease settings.^38^ However, in the context of respiratory fungal infection, the mechanistic basis of spore uptake and implications for disease pathogenesis remain unknown.

Using our established single-cell approaches to study AECs infected with *Af*, we set out to understand the role of fungal spore uptake in the pathogenesis of human disease. Here, we demonstrate, for the first time in animal models, that *Af* spores are internalized and killed by AECs. Using primary human AECs from healthy and COPD donors, we also demonstrate that lung disease significantly impacts *Af* uptake, suggesting that aberrancies in uptake might promote fungal infection of diseased lungs. We conclude that human AECs exert potent control over inhaled *Af* spores, providing a frontline defense against in-host germination and invasive disease. A more comprehensive understanding of AEC uptake could facilitate new treatments and limit respiratory damage.

## Results

### A549 cells efficiently kill internalized *Af* spores upon *in vitro* infection

**Figure 1:**
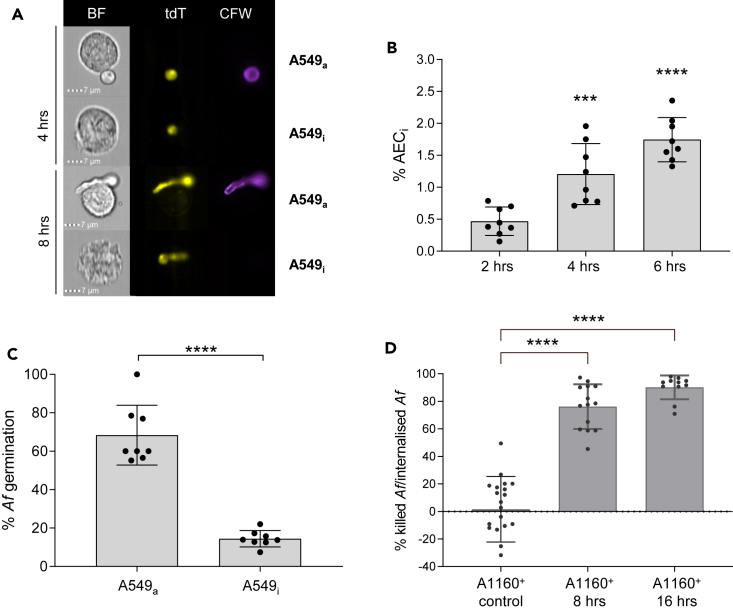
Single-cell analysis of *Af-*AECs interactions indicate that, while viability of internalizing A549 cells is unaffected, internalized *Af* is rapidly killed by A549 cells Infection of A549 monolayers with A1160^+/tdT^ (*Af*^tdT^) (MOI 0.5). (A) IFC exemplary panels showing A549 cells which have *Af* on their surface (A549_a_) or inside (A549_i_) (4 h post-infection) and differential *Af* germination on or inside A549 cells (8 h post-infection). (B) Quantification of A549_i_ after infection for the indicated time points. Percentage of A549_i_ is derived on 2000 total cell analyzed. (C) Percentage of *Af* germination in A549_a_ and A549_i_ (8 h post-infection, unpaired t test relative to uninfected control). (D) Percentage of dead intracellular *Af* in A549_i_ relative to internalized *Af* (8 and 16 h post-infection). For (B)–(D), all experiments were carried out in biological triplicates with technical duplicates/triplicates. (B) and (D) were analyzed with non-parametric Kruskal-Wallis test with Dunn’s multiple comparisons test relative to uninfected control (not shown) for (B) and to the respective *Af*^tdT^ spore sorting control (n = 3–5 pools of 100 AEC_i_ for each sample in biological triplicates) for (D). ∗∗∗∗*p* ≤ 0.0001, ∗∗∗*p* ≤ 0.001, ∗∗*p* ≤ 0.01, and ∗*p* ≤ 0.05. Data are represented as mean ± SD. Scale bar, 7 μm.

In order to perform a high-throughput parameterization of rate, stoichiometry, and outcomes of *Af* uptake by AECs, we previously developed a multiplexed assay, which combines differential fluorescence staining of red-fluorescent tdTomato (tdT)-expressing strains with the cell impermeant fluorescent stain Calcofluor White (CFW) and imaging flow cytometry (IFC).^39^ This multiplex assay allows the visualization, quantification, and analysis of *Af*-AEC interactions at the single-cell level and differentiates AECs which have internalized *Af* spores (AEC_i_: CFW^−^ tdT^+^) from AECs attached to fungal elements (AEC_a_: CFW^+^tdT^+^) (Figure 1A). The inclusion of Annexin-V-fluorescein isothiocyanate (FITC) (Anx-FITC) and TO-PRO-3 (TO-PRO3) differentiates two modes of host cell death, namely apoptosis (Anx^+^) and necrosis (TO-PRO3^+^). As we previously published^39^ for the tdT-expressing strain ATCC46645^tdT^,^40^ we first confirmed that A549 cells are also able to rapidly internalize spores of another tdT-expressing strain, namely, the A1160^+/tdT^ isolate (Figures 1A, 1B, and S1) without undergoing significant apoptosis or necrosis as a consequence of uptake (not shown). IFC-mediated enumeration of AECs internalizing the A1160^+/tdT^ isolate from 2 to 6 h post-infection showed a rapid, significant, and sustained spore uptake during *in vitro* infection, whereby the percentage of AEC_i_ at 6 h of infection (1.75%, *n* = 34.9 ± 6.9 cells from 2,000 AECs surveyed) was 4 times higher than that at 2 h (0.5%, *n* = 9.4 ± 4.5 cells from 2,000 AECs surveyed) (Figure 1B).

While most of the *Af* elements attached onto AEC_a_ had germinated within 8 h of infection (68.4% ± 15.59%, *n* = 171), most of the *Af* spores internalized by AEC_i_ were ungerminated or delayed for germinative growth (14.5% ± 4.2%, *n* = 696) (Figure 1C). Viability of internalizing A549 cells was largely unaffected, but the quantification of the percentage of *Af* germination for AEC_a_ and AEC_i_ showed that, after 8 h of infection, most of the *Af* spores internalized by A549 cells were ungerminated or delayed for germinative growth in comparison to their extracellular counterparts closely attached to AECs (Figure 1D).

The impairment of germination in internalized spores (Figure 1C) suggested that *Af* might be killed upon uptake. However, likely due to the use of different experimental settings, current studies show contrasting findings regarding the intracellular fate of internalized *Af* spores within A549 cells. Uptake was reported to lead to either significant *Af* killing or, more rarely, intraphagosomal occupancy and persistence.^34^^,^^35^^,^^36^^,^^41^^,^^42^^,^^43^^,^^44^ Thus, in order to measure *Af* viability upon uptake in our experimental system, fluorescence-activated cell sorting (FACS) was used to sort pools of 100 internalizing A549 cells after infection with A1160^+/tdT^ for 8 and 16 h. AEC_i_ pools were subsequently lyzed to release the intracellular fungal elements and to perform fungal viability counts. Viability counts of internalized *Af* spores revealed a rapid killing of the intracellular fungal population, whereby, by 8 h post-infection, 76.2% (±16.23) of the internalized A1160^+/tdT^ fungus was killed by AEC_i_ (Figure 1D). Fungal killing upon uptake by A549 cells was almost complete by 16 h of infection, whereby 90.2% (±8.6) of the internalized A1160^+/tdT^ fungal population was not retrieved in the viable counts. Taken together, our findings demonstrate a striking ability of A549 cells to quell fungal spores upon uptake, providing evidence that AECs might play a critical contribution to killing of inhaled *Af* spores by means of their phagocytic activities.

### *Af* is internalized and killed by AECs during infection in mice

**Figure 2:**
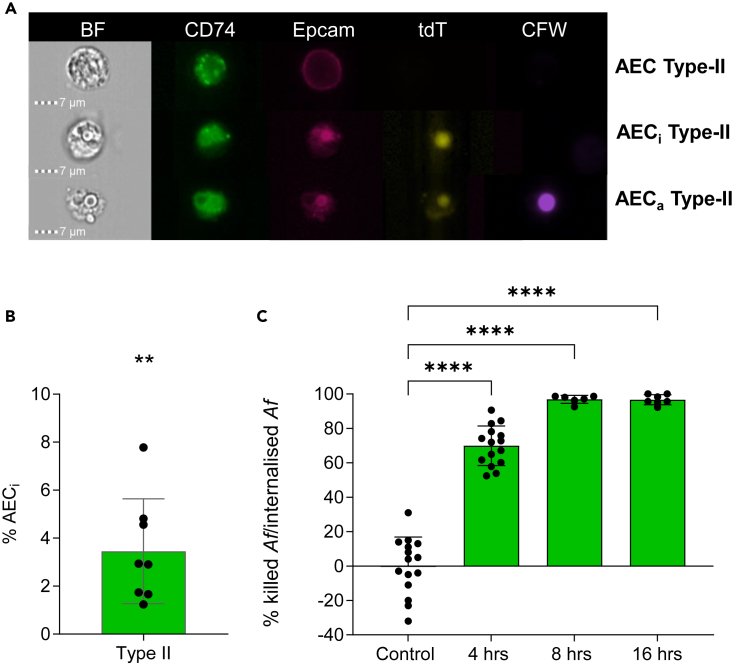
Murine type-II AECs harvested directly from infected leukopenic mice ingest and kill *Af* spores during infection (A) IFC exemplary panels showing type-II (CD74^+^ EpCam^+^) AECs, which have *Af* on their surface (AEC_a_) or inside (AEC_i_) and differential *Af* germination on or inside type-II AECs (8 h post-infection). (B) Percentage of type-II (CD74^+^ EpCam^+^) AECs internalizing *Af* when surveying 800 cells. Leukopenic mice were infected with 10^8^ spores of ATCC46645^tdT^ for 8 h. IFC-acquisition was carried out once for the 2 uninfected lungs, while 4 technical replicates were acquired from the 2 pools of 3 lungs obtained from 8-h infected mice. Data were analyzed with non-parametric Kruskal-Wallis test with Dunn’s multiple comparisons test relative to uninfected control (not shown). (C) Percentage of dead intracellular *Af* in murine AEC_i_ relative to internalized *Af* at 4, 8 and 16 h post-infection with ATCC46645^tdT^. Significance for each time-specific sample is shown relative to the 100 *Af*^tdT^ spore sorting control (n = 1–5 pools of 100 AEC_i_ for 3 pools of 5 lungs obtained from 4, 8 and 16 h infected mice, Kruskal-Wallis test with Dunn’s multiple comparisons). ∗∗∗∗*p* ≤ 0.0001, ∗∗∗*p* ≤ 0.001, ∗∗*p* ≤ 0.01, and ∗*p* ≤ 0.05. Data are represented as mean ± SD. Scale bar, 7 μm.

While multiple studies have reported spore uptake by immortalized and/or primary AECs in *in vitro* infection systems and *ex vivo* organ culture models,^27^^,^^28^^,^^29^^,^^30^^,^^31^^,^^32^^,^^33^^,^^34^^,^^37^^,^^45^^,^^46^ no compelling evidence of *in vivo* spore uptake by AECs has been published so far. In order to test if *Af* spores are internalized by AECs during mammalian infection, we initially optimized the isolation of type-II AECs from infected mice (Figures 2A and S3). To maximize the resolution of *Af*-AEC complexes and limit the interference of phagocytic activities by innate immune cells, an established leukopenic mouse model was used^47^ and mice were infected with the tdT-expressing strain ATCC46645^td^ ^40^ for 8 h. The multiplex IFC platform was modified and optimized to quantify type-II epithelial cell subpopulations based on antibody-mediated labeling of epithelial cellular adhesion molecule (EpCam) and CD74. While EpCam is routinely used as a generic marker for epithelial cells,^48^^,^^49^^,^^50^ CD74 has been demonstrated to be excellent marker to isolate type-II AECs (CD74^+^ EpCam^+^)^51^ (Figure 2A). From digested lung tissue, harvested directly from infected leukopenic mice 8 h after infection, 3.5% (±2.2) of the murine type-II AECs (of 800 cells surveyed) were found to internalize *Af* using IFC (Figure 2B).

To measure *Af* viability upon uptake by murine primary AECs obtained from infected dissociated lung tissue, FACS was used to sort pools of 100 AEC_i_ after infection with ATCC46645^tdT^ for 4, 8, and 16 h. AEC_i_ pools were subsequently lyzed to release the intracellular fungal elements and to perform fungal viability counts (Figure 2C). Viability counts of internalized *Af* spores revealed a rapid killing of the intracellular fungal population by AECs, whereby, by 4 h post-infection, 69.9% (±11.5) of the internalized fungus was killed by type-II AECs, and almost complete killing (>97% ± 2.3) was measured after 16 h of infection (Figure 2C). In conclusion, our single-cell approach, applied directly to infected mouse lungs, demonstrated that *Af* spores are internalized and killed by AECs during whole-animal infection.

### Lung disease significantly impacts uptake of fungal spores

**Figure 3:**
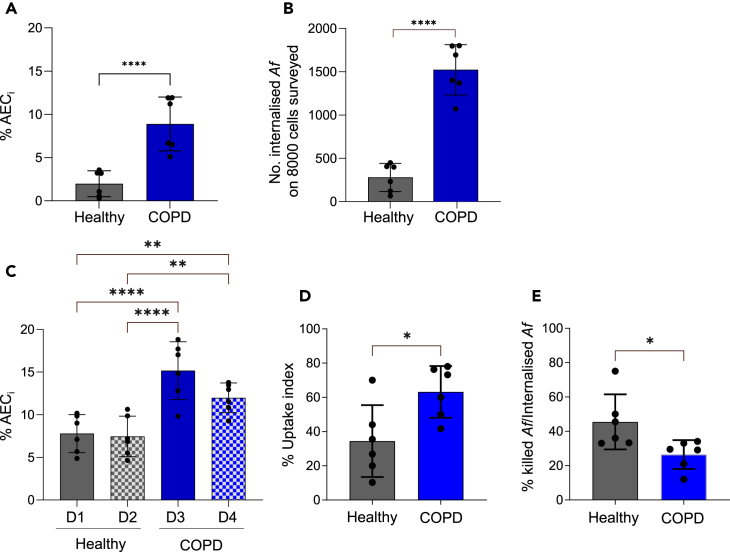
Single-cell comparison of uptake and intracellular killing by primary human AECs indicate that COPD-derived AECs are aberrant in their interaction with fungal spores (A) Percentage of AEC_i_ when surveying 8,000 commercially derived cells infected with A1160^+/tdT^ (*Af*^tdT^) (MOI 0.5) by IFC. (B) Number of *Af* spores internalizzed when surveying 8,000 AEC in (A) by IFC. (C) Percentage of AEC_i_ on 8,000 locally sourced cells surveyed by IFC. (D and E) (D) Uptake index by and (E) percentage of intracellular killing within commercially derived AEC_i_ as quantified by live-cell microfluidic imaging. With the exception of (C), AEC tested were purchased from Lonza. For (C), cells were obtained from locally sourced lung resections from two healthy donors (D1 and D2) and two COPD sufferers (D3 and D4) via collaboration with the MFT ManARTS Biobank. For (A)–(D), infections of epithelial monolayers with *Af* were carried out for 6 h, while in (E) infections were carried out for 20 h. All experiments were conducted in biological duplicates and technical triplicates (ordinary 1-way ANOVA with Holm-Sidak’s multiple comparisons for A–C and unpaired t test for D and E). ∗∗∗∗*p* ≤ 0.0001, ∗∗∗*p* ≤ 0.001, ∗∗*p* ≤ 0.01, and ∗*p* ≤ 0.05. Data are represented as mean ± SD.

On the premise that epithelial activities provide a potent means of antifungal defense *in vivo*, we hypothesized that uptake by AECs would also happen in human primary AECs and might be impaired in patients susceptible to fungal lung disease, such as COPD sufferers. Using IFC on commercially available AECs from both healthy and COPD donors, we demonstrated that primary human AECs do indeed internalize *Af* A1160^+/tdT^ spores at 6 h post-infection, at similar levels to A549 cells (2.0%, *n* = 158.5 ± 119.6 cells on 8,000 cells surveyed) (Figure 3A). COPD-derived AECs were found to internalize significantly more *Af* than their healthy counterpart, whereby the percentage of COPD-derived AEC_i_ at 6 h of infection (8.9%, *n* = 711.3 ± 249.4 cells on 8,000 cells surveyed) was over 4 times higher than the percentage of healthy-derived AEC_i_ (Figure 3A). Accordingly, the overall *Af* count within all AECs surveyed (*n* = 8,000) was significantly higher for infections of COPD-derived AECs, with 1,522 (±290.9) A1160^+/tdT^ spores internalized by COPD-derived AECs and only 279.3 (±161.9) spores internalized by healthy AECs (Figure 3B). Taken together, these results indicate that COPD significantly impacts uptake of fungal spores. To further corroborate this finding, we extracted and challenged primary human AECs, sourced from the respiratory clinics of the Manchester University NHS Foundation Trust (MFT). Locally sourced AECs derived from two donors with and without COPD were subject to IFC-mediated quantification of spore uptake (Figure 3C) revealing similar findings to the data obtained from commercially available AECs. COPD-derived AECs internalized significantly more A1160^+/tdT^ spores (15.2% ± 3.4%, *n* = 1,214 ± 271.2 cells for D3 and 12% ± 1.7%, *n* = 958.3 ± 139.7 cells for D4 on 8,000 cells surveyed) than healthy AECs (7.8% ± 2.2%, *n* = 622.6 ± 178.4 cells for D1 and 7.5% ± 2.4%, *n* = 596.0 ± 189.7 cells for D2 on 8,000 cells surveyed) (Figure 3C).

Due to the limited number and lifespan of primary human AECs, microfluidic live-cell semi-automated imaging of primary epithelial monolayers from commercially available AECs exposed to fungal challenge was opted to determine if the intracellular fate of internalized *Af* was also impacted by COPD (Figures 3D and 3E). As the fluorescent molecule mScarlet is more rapidly quenched upon intracellular killing^42^^,^^52^ than tdT, we generated and used mScarlet-expressing strains, A1160^+/*mScar*^, to trace fungal viability within AECs (Figure S2). In support of our previous IFC observations (Figures 3A–3C), the uptake index (% internalized *Af*/total *Af*) of COPD-derived AECs after 6 h of infections was twice as much (63.2% ± 15.1%) as that of healthy AECs (34.5% ± 21.0%) (Figure 3D). Most importantly, in agreement with the central hypothesis that the respiratory epithelium plays a critical role in pathogen protection, we demonstrated that the increased uptake of fungal spores in COPD-derived AECs (Figures 3A–3D) correlated with the inability of gorging COPD-derived AECs to quell intracellular *Af* as efficiently as healthy primary AECs (Figure 3E). After quantification of uptake at 6 h post-infection, the intracellular fate of internalized A1160^+/mSca^ (Figure S2) was followed in real time for a further 14 h. Lack of germination and quenching of mScarlet fluorescence in internalized A1160^+/mSca^ was measured as a proxy of intracellular killing and was significantly lower (around half) within COPD-derived AECs (26.5% ± 8.4%) than healthy AECs (45.5% ± 16.0%) (Figure 3E). Notably, killing percentages of mScarlet-expressing *Af* in the microfluidic live-cell experiments with primary human AECs (Figure 3E) were much lower than killing percentages of tdT-expressing *Af* upon FACS-mediated sorting of immortalized or primary murine AEC_i_ (Figures 1D and 2C). This suggests that mScarlet quenching within AECs directly correlates, but it is not equivalent to loss of *Af* viability and highlights the needs of developing novel, more efficient, direct reporters of *Af* viability within host cells. Nevertheless, taken together, these findings suggest that altered AEC responses to fungal challenge represent a significant risk factor for *Aspergillus*-related diseases in COPD.

## Discussion

Following inhalation, the respiratory epithelium is the initial point of contact for a multitude of respiratory pathogens, which subsequently elicit highly varied and multifaceted host responses. Entry into host mucosae has been conventionally recognized as a pathogenic strategy exploited by microbes to thrive within the host to further perpetuate infection; however, growing evidence demonstrates that the airway epithelium also plays a crucial, and likely underestimated, role in host defense by opsonizzing, ingesting, and quelling inhaled microorganisms and initiating innate responses.^38^ As most *Aspergillus*-related disease manifestations initiate with the inhalation of fungal conidia and eventually lead to the destruction of the pulmonary parenchyma, understanding the AEC-driven mechanisms of microbial clearance is of pivotal importance.

Using *in vitro* and *ex vivo* infection models, we and others have previously demonstrated that *Af* uptake is one of the earliest events following spore adhesion to airway epithelial cells, with approximately 30%–50% of adherent *Af* internalized by bronchial or type-II AECs in a concentration- and time-dependent manner.^27^^,^^28^^,^^29^^,^^30^^,^^53^^,^^54^ In *in vitro* infections, uptake by airway epithelial cells can lead to *Af* killing or, more rarely, intraphagosomal occupancy.^34^^,^^35^^,^^36^^,^^41^^,^^42^^,^^43^^,^^44^ Population-scale analyses further indicate that phagosomal acidification results in killing of 97%–98% of the internalized conidia within 12–24 h, while ∼2%–3% of the intracellular *Af* remain viable and in a third of the cases can eventually germinate by 36 h, without lysis of the host cells.^31^ Our study and those of others indicate that airway epithelial cells potently neutralizze internalized spores (Figure 1), strongly supporting a curative role for epithelial activities.^31^^,^^37^^,^^55^

While spore uptake has been reported for immortalized bronchial and AECs in *in vitro* infection systems and *ex vivo* organ culture models,^25^^,^^27^^,^^29^^,^^30^^,^^31^^,^^32^^,^^33^^,^^37^^,^^45^^,^^46^ no compelling data had yet supported the occurrence or physiological relevance of *in vivo* spore uptake by AECs. Applying our flow cytometric experimental pipeline to analyze *Af*-AECs interactions directly from infected murine lungs, we were able to demonstrate *Af* spore uptake by AECs and the relevance of this process during murine infection. To the best of our knowledge, this represents the first published evidence that *Af* uptake occurs, during mammalian infection, and that AECs possess a striking fungicidal activity (Figure 2), which might contribute to killing of inhaled *Af* spores during infection.

While the role of AEC-mediated clearance in an immunocompetent host remains to be clarified, suboptimal killing of internalized *Af* spores might result in AECs serving as a fungal reservoir for latent occupation and immune evasion in an immunocompromized host.^34^^,^^35^^,^^36^ Indeed, our findings indicate that spore uptake by AECs is likely to range from a useful contribution to neutralizing low-level exposure in health to a driver of invasive growth and pathogenesis^25^^,^^27^^,^^31^^,^^37^ where intracellular killing is not possible, as during disease.^37^ We demonstrate that COPD-derived AECs internalized significantly more *Af* than their healthy counterparts and, more importantly, gorging COPD-derived AECs were unable to quell intracellular *Af* as efficiently as healthy primary AECs (Figure 3). IA constitutes a significant complication of COPD with recent estimates indicating that nearly 3.9% of COPD patients admitted to hospital annually develop IA, resulting in 540,451–977,082 predicted deaths annually.^6^^,^^7^ However, our current understanding of the mechanisms underlying the increased susceptibility of these patients to respiratory infections is limited due to the inherent heterogeneity of COPD and the lack of standardized and high-throughput *in vitro* and *in vivo* models of disease.^56^^,^^57^^,^^58^^,^^59^ More experiments are currently ongoing to increase our cohort size with a view to i) establish donor-dependent variance of spore uptake and intracellular killing by AECs from patients with different COPD severities and ii) understand if altered protective activities against *Af* in COPD-derived AECs concur with dysregulation of host cell death and concomitant immune responses. While the underlying mechanism for increased uptake but reduced killing remains so far elusive, our findings suggest that altered AEC responses to fungal challenge may represent a risk factor for IA in COPD.

Our single-cell approach unmasked the complexity of the *Af*-AECs interactions with respect to the relevance of *Af* uptake by AECs in the context of mammalian disease and susceptibility to fungal infection. These observations and the powerful technologies we developed to obtain these are currently driving our further investigations into the molecular, transcriptional, and immunological basis of the *Af*-AEC interaction *in vitro*, *in vivo,* and in primary AECs. Such studies are aimed to unravel how protective *Af*-AECs interactions contribute to mucosal tissue homeostasis and pathogen clearance upon *Af* inhalation and can become dysregulated in the settings of pre-existing respiratory disease, thereby driving *Aspergillus*-related diseases. An in-depth understanding of how the lung balances mucosal tissue homeostasis and pathogen clearance via antimicrobial epithelial activities has major clinical implications as it would aid the identification of immunomodulators to facilitate treatment and limit respiratory damage against respiratory infections caused by not only *Af* but also other respiratory pathogens of fungal and non-fungal etiology.

### Limitations of the study

Our unique single-cell approach unmasked the intricate nature of *Af*-AECs interactions and the importance of *Af* uptake by AECs in the context of mammalian infection and susceptibility to fungal infection. We trained the experimental workflow on well-documented assay systems (A549 cells)^39^ such that the workflow could subsequently be transposed to study host-pathogen interactions from *ex vivo* primary cells and during murine infection.

This study focused on understanding the relevance of alveolar epithelial activities during disease; however, further investigations are required and currently ongoing to clarify the role and mechanisms underpinning epithelial-mediated antifungal potency in both health and disease, using physiologically relevant infection *in vitro* and *in vivo* systems. Our results highlighted that dysfunctional epithelial activities might have a role in increasing susceptibility of COPD sufferers to IA. However, a more extensive sampling of healthy or COPD individuals is currently in progress to establish donor-dependent differences in *Af*-AEC interactions and outcomes in patients with different COPD severities. Regardless, our findings point toward AECs as important initial participants in *Af*-host cell interactions at the alveolar interface; thus, understanding how AECs contribute to the balance of mucosal tissue health and clearance of *Af* following inhalation and how this may become disrupted in the context of pre-existing respiratory conditions holds significant clinical implications.

## STAR★Methods

### Key resources table

**Table:** 

REAGENT or RESOURCE	SOURCE	IDENTIFIER
**Antibodies**		

PE/Cyanine7 anti-human CD326 (EpCAM) Antibody	Biolegend	Cat#324221
FITC anti-human CD74 Antibody	Santa Cruz	Cat#sc-6262
Dynabeads® CD45	ThermoFisher	Cat#11153D
Dynabeads® CD31	ThermoFisher	Cat#11155D
Purified anti-mouse CD16/32 Antibody	BioLegend	Cat#101302
PE/Cyanine7 anti-mouse CD326 (Ep-CAM) Antibody	Biolegend	Cat#118215
PE anti-mouse Podoplanin Antibody	BioLegend	Cat#127407
FITC anti-human CD74 Antibody	Santa Cruz	Cat#sc-19627

**Biological samples**		

Primary human alveolar epithelial cells	Obtained in this study from tissues from Manchester University NHS Foundation Trust (MFT)	N/A

**Chemicals, peptides, and recombinant proteins**		

Liberase™ TL Research Grade	Sigma-Aldrich	Cat#05401020001
DNAase (Deoxyribonuclease I from bovine pancreas)	Sigma-Aldrich	Cat#D5025-150KU
Dispase® II (neutral protease, grade II)	Sigma-Aldrich	Cat#04942078001
Calcofluor white (CFW, Fluorescent Brightener 28 disodium salt solution)	Sigma-Aldrich	Cat#910090

**Critical commercial assays**		

GeneArt™ Seamless Cloning and Assembly Kit	ThermoFisher	Cat#A13288
Southern blotting: Anti-Digoxigenin-AP, Fab fragments	Roche	Cat#11093274910
Southern blotting: CDP-Star, ready-to-use	Roche	Cat#12041677001
Southern blotting: PCR DIG Probe Synthesis Kit	Roche	Cat#11636090910
Southern blotting: DIG Easy Hyb	Roche	Cat#11603558001
Annexin V-FITC Apoptosis Kit	Biovision	Cat#K101-100
TO-PRO-3 Iodide (642/661), 1 mM Solution in DMSO	ThermoFisher	Cat#T3605

**Experimental models: Cell lines**		

Human pulmonary carcinoma epithelial cell line A549	ATCC	Cat#CCL-185
Primary Human small airway epithelial cells	Lonza	Cat#cc-2547 (Batch number TAN 29807) and cc-2934 (Batch number TAN 23484)

**Experimental models: Organisms/strains**		

*A. fumigatus* strain A1160^+^	Rizzetto et al., 2013^60^	N/A
*A. fumigatus* strain ATCC46645t^dT^	Lother et al., 2014^40^	N/A
*A. fumigatus* strain A1160^+/tdT^	This study	N/A
*A. fumigatus* strain A1160^+/mScar^	This study	N/A
Mouse: Male CD1 mice (18–22 g)	Charles River	N/A

**Oligonucleotides**		

mScarlet1c CACCGTTTATGGTGAGCAAGGGCGAGGCAGTG	This study	N/A
mScarlet2c TGGCGTTTCTACTTGTACAGCTCGTCCATGCCGCC	This study	N/A
pSK536_1 TGTACAAGTAGAAACGCCATGTCTATCTTCGAGTA	This study	N/A
pSK536_2 CTCACCATAAACGGTGATGTCTGCTCAAGCGG	This study	N/A
tdTomato1 CAGTTCATGTACGGCTCCAA	This study	N/A
tdTomato2 AGATGGTCTTGAACTCCACCA	This study	N/A
tdTomato3 GTAACTACGCTCAACGTGTT	This study	N/A
tdTomato4 CTTCCTGTTGATGGAATGG	This study	N/A
PtrA_SB1 GGATAGGGGCGAACTTGAACT	This study	N/A
PtrA_SB2 TTTGGCTGGACTCTCACAAT	This study	N/A
mScarlet1 TCCCCTCAGTTCATGTACGG	This study	N/A
mScarlet 2 CTTGTACAGCTCGTCCATGC	This study	N/A
His2A1 TTCACCTGATTCAGCTGATTG	This study	N/A
His2A2 TTCACCTGATTCAGCTGATTG	This study	N/A
PtrA_SB1 GGATAGGGGCGAACTTGAACT	This study	N/A
PtrA_SB2 TTTGGCTGGACTCTCACAAT	This study	N/A

**Recombinant DNA**		

Plasmid tdTomato pSK536	Lother et al., 2014^40^	N/A
Plasmid mScarlet pSK536^mScarlet^	This study	N/A
pmScarlet_C1	Addgene	Cat#85042

**Software and algorithms**		

Fiji software	Schindelin et al., 2012^70^	https://imagej.nih.gov/ij/
BD FACSDIVA v.8.0.1 software	BD Biosciences	N/A
FlowJo v.10 software	BD Biosciences	N/A
IDEAS® v.6.2 software	Merk	N/A
ONIX v.5.0 software	Millipore	N/A
Nikon Elements v.4.2 acquisition software	Nikon	N/A
GraphPad Prism v.8	Dotmatics	https://www.graphpad.com/features

**Other**		

DMEM High Glucose with L-Glutamine and Sodium Bicarbonate/without Sodium Pyruvate	Sigma-Aldrich	Cat#D5796
Fetal bovine serum (FBS)	Sigma-Aldrich	Cat#F9665
Penicillin/streptomycin cocktail	Sigma-Aldrich	P0781
Trypsin-EDTA solution	Sigma-Aldrich	Cat#T3924
Small Airway Epithelial Cell growth medium	Promocell	Cat#C-21070
Dulbecco’s Phosphate Buffered Saline (PBS)	Sigma-Aldrich	Cat#D8537
Red blood Cell Lysis	Sigma-Aldrich	Cat#R7757
DMEM/F-12	Gibco™	Cat#11320033
Percoll	MP Biomedicals	Cat#02195369-CF
Hanks′ Balanced Salt solution (HBSS)	Sigma-Aldrich	Cat#H9269
2-well chambered cover glasses	Ibidi	Cat#IB-80287
Aspergillus complete media (ACM)	Pontecorvo et al., 1953^68^	N/A
Aspergillus minimal media (AMM)	Pontecorvo et al., 1953^68^	N/A
FluoroBrite™ DMEM	Gibco™	Cat#A1896702
CellASIC M04S-03 Microfluidics plates	Millipore	Cat#M04S-03-5PK
Cellasic ONIX2 Microfluidic System	Millipore	Cat#CAX2-S0000
Cellasic ONIX2 Manifold	Millipore	Cat#CAX2-MBC20
BD LSRFortessa system	BD Biosciences	N/A
BD Influx Cell Sorter	BD Biosciences	N/A
ImageStream®X Mark II Imaging Flow Cytometer	Merk	N/A
Nikon TiS Eclipse microscope A	Nikon	N/A

### Resource availability

#### Lead contact

Further information and requests for resources and reagents should be directed to and will be fulfilled by the lead contact, Dr Margherita Bertuzzi (margherita.bertuzzi@manchester.ac.uk).

#### Materials availability

The plasmids pSK536^mScarlet^ and strains Af^tdT^ and Af^mScar^ (in the A1160^+^ genetic background) generated in this study have been deposited in the MFIG collections and will be made available to the community upon request to the lead contact.

#### Data and code availability

• Data: All data reported in this paper will be shared by the lead contact upon request.
• Code: This paper does not report original code.
• All other items: Any additional information required to reanalyse the data reported in this paper is available from the lead contact upon request.

### Experimental model and subject details

#### Epithelial cell culture

The epithelial cell line used in this study was the human pulmonary carcinoma epithelial cell line A549 (American type culture collection, CCL-185). A549 cells were maintained at 37°C, 5% CO_2_ in supplemented DMEM (sDMEM, 10% fetal bovine serum (FBS), 1% penicillin/streptomycin cocktail). Primary human AECs were purchased from Lonza (Healthy = cc-2547, Batch number TAN 29807 [67 Y Female, no diabetes, no smoker] and COPD = cc-2934, Batch number TAN 23484 [57 Y Female, no diabetes, smoker]) following isolation from the distal portion of the human respiratory tract in the 1 mm bronchiole area. Donors were matched as much as possible in terms of age, sex and smoking status in order to minimise differences due to these factors. Alternatively, primary human AECs (see below) were isolated and maintained in culture from lung resections obtained via the Manchester Allergy, Respiratory and Thoracic Surgery (ManARTS) Biobank from the Manchester University NHS Foundation Trust (MFT), which is authorised by the National Research Ethics Service (NRES) to release samples to researchers. Primary human AECs were maintained at 37°C, 5% CO_2_ in Small Airway Epithelial Cell growth medium (SAEC, Promocell). All cells were used for infections when at ≥ 90% confluence. All primary cells, whether of commercial or local origin, were checked for purity via flow cytometry following staining with the following antibodies as from manufacturers specifications: EpCAM-PE-Cy7 (Biolegend, 324221) for epithelial cells and CD74-FITC (Santa Cruz, sc-6262) for type-II AECs.^51^ Fluorescence of AECs measured using a BD LSRFortessa system (BD Biosciences) and the BD FACSDIVA 8.0.1 software. The results were analyzed using the FlowJo v10 software. Only monolayers with ≥90% alveolar type-II cell content (Epcam^+^ CD74^+^) where used for *in vitro* infections. For the microscopy experiments, 3 × 10^4^ A549 cells were seeded in 2 mL of sDMEM in 2-well chambered cover glasses (Ibidi). For flow cytometric analysis, 3 × 10^4^ A549 cells were seeded in 2 mL sDMEM in 6-well plates. For microfluidic experiments, harvested cells were resuspended in sDMEM at the concentration of 2.5 x10^6^ cells/mL. CellASIC M04S-03 Microfluidics plates (Merck) were equilibrated and prepared as described by the manufacturers.

#### Primary human AECs from lung resections

Lung resections were obtained from the ManARTS Biobank from donors with and without pre-existing lung disease undergoing surgery. Tissues from donors on immune modulatory therapy or with the following diseases were excluded: rheumatoid arthritis, other inflammatory diseases, other autoimmune diseases and tuberculosis. Once passed the exclusion criteria, donors were defined as healthy if showing i) lung function with Forced Expiratory Volume in first second (FEV1)/Forced Viral Capacity (FVC) > 0.7 and FEV1 > 80%, ii) no mention of COPD or emphysema in their hospital records and iii) no diagnosed Type 2 diabetes. COPD donors were classified as definite if i) FEV1/FVC <0.7 and COPD box ticked and ever smoker, ii) FEV1/FVC <0.7 and emphysema on CT scan and ever smoker or iii) FEV1/FVC <0.7, ever smoker and positive symptoms or probable if FEV1/FVC <0.7, ever smoker but nil else on proforma. Post-surgery diagnostic analysis of the patient samples and records was used to confirm moderate COPD (FEV1/FVC was between 55 and 70% for the COPD donors described); however, no other information about sex or age are available. Other investigators may have received tissue from the same subjects.

#### Fungal strains

For *in vitro* experiments, the *Af* strains used was A1160^+^,^60^ which was genetically modified to constitutively express the red fluorescent tdTomato (554/581 nm)^61^ and mScarlet (569/594 nm).^52^ The choice of this isolate was driven by the availability of a library of null mutants in this genetic background, which will be used for our further investigations into the molecular, transcriptional and immunological basis of the *Af*-AEC interaction.^62^ For murine infections, the published tdTomato-expressing strain ATCC46645^tdT^ ^40^, a derivative of the clinical isolate ATCC46645,^63^ was used. As tdTomato has high photostability and low pH sensitivity,^61^ *Af*^*tdT*^ strains retain fluorescence upon intracellular killing, permitting high-throughput assessment of uptake rates and stoichiometry via IFC^39^ and FACS. In contrast, mScarlet has moderate acid sensitivity and is more rapidly quenched upon intracellular killing^52^; therefore, *Af*^*mScar*^ strains represent an ideal tool to trace fungal viability within AECs via microscopy.

### Methods details

#### Isolation of primary human AECs from lung resections

For the isolation of primary human AECs from lung resections, modifications of the published protocols were adopted.^64^^,^^65^^,^^66^^,^^67^ 3–5 g of resected lung tissue was finely cut up to be placed into 8 mL of freshly made digestion buffer, containing 0.4 U/mL Liberase (Sigma) + 160 U/mL DNAse (Sigma) + 0.1 mg/mL Dispase (Sigma). After incubation for 45 min at 37°C, tissue digestion was stopped by adding 10 mL of cold Stop solution (PBS +2 mM EDTA +2% FCS). Digested lung tissue was filtered serially through a 100 μm, 70 μm and 40 μm cell strainer and for each filtration step, the cell strainers were rinsed with 10 mL of Stop solution. The cell suspension obtained was filtered at 400 g, room temperature for 5 min. After centrifugation, the supernatant was discarded and the pellet was resuspended in 10 mL of cold 1X Red Blood Cell Lysis (RBC) buffer. Samples were vortexed and incubated in the dark for 10 min at room temperature. 2 mL of cold PBS were added and samples were centrifuged at 700 g, 4°C for 10 min. After centrifugation, pellets were resuspended in 10 mL of adhesion medium for macrophages and fibroblasts (22.5 mL DMEM/F-12, 22.5 mL unsupplemented SAEC growth medium, 10% mL FBS and 160 U/mL DNase I). Cells were transferred to a T75 tissue culture flask and incubated at 37°C for 60–90 min. This step was repeated twice more and AECs not adhering were collected and centrifuged at 300 g for 10 min at room temperature. Following centrifugation, the pellets were resuspended in 3 mL DMEM/F-12 and the cell suspension was layered on a 1.040–1.089 g/mL discontinuous Percoll (MP Biomedicals) gradient. Centrifugation at 300 *g* for 20 min at 4°C using a swing out rotor was used to generate an enriched layer of AECs at the interface between the two layers of Percoll gradients. The enriched layer of AECs was transferred to a 15 mL centrifuge tube containing 13 mL HBSS and centrifuged at 300 g for 10 min at room temperature. Following centrifugation, pellets were resuspended in 1 mL of Dynabuffer Isolation Buffer 2 for magnetic bead separation with Dynabeads CD45 and Dynabeads CD31 (ThermoFisher) as from manufacturer’s instructions to remove any possible contamination of macrophages and endothelial cells respectively. Unbound AECs were transferred for culturing in 5 mL of supplemented SAEC growth medium and maintained in culture for up to 2 months.

#### Fungal strains preparation

*Af* strains were cultured at 37°C in *Aspergillus* Complete Media (ACM)^68^ and for the preparation of spore suspensions, spores were harvested, filtrated through Miracloth and centrifuged at 2000 g for 5 min. After counting, a 10^6^ spores/mL suspension was prepared in sDMEM. The inoculum concentrations were verified performing viable counts of serial dilutions on ACM plates in duplicate. Cells were infected with 200 μL of the 10^6^ spore/mL suspension following the replacement of cell media with 2 mL of fresh sDMEM to obtain a MOI 0.5 (with 10^5^ AECs in a >90% confluent monolayers). The infected cells were incubated at 37°C, 5% CO_2_ for the indicated hours. While previous titration of fungal challenge has indicated linearity in uptake rates,^39^ the chosen concentration of conidia elicits <40% of epithelial detachment^27^ and necrosis (via LDH release)^26^ within 16 and 24 h respectively, thereby permitting analysis of *Af*-AECs complexes within the time frame proposed. Time-points for the analysis were chosen based on previous experimental optimisation *in vitro,* which shown that 6 and 12/16 h post-infection represent the highest rate of spore uptake and the highest rate of intracellular killing.

#### Generation and verification of tdTomato- and mScarlet-expressing *Af* strains

The plasmid pSK536 was used for the targeted insertion of the tdTomato fluorophore into the *Af* genome.^40^ In pSK536, tdTomato is under the control of the constitutively expressed *A. nidulans* glyceraldehyde-3-phosphate dehydrogenase gene (ANIA_08041) promoter (*gpdA*) and is targeted at the *Af* region intergenic to the AFUA_3G05360 and AFUA_3G05370, where the terminator region of the *Af* histone 2A locus (his2A^t^) is placed. The *A. oryzae* pyrithiamine resistance marker (*ptrA*) marker is present in the plasmid for selection in fungal cells. Using the GeneArt technology (ThermoFisher) as from manufacturer’s specifications, the plasmid pSK536 was modified into pSK536^mScarlet^ for the targeted insertion of the mScarlet fluorophore into the *Af* genome. mScarlet was amplified from the plasmid pmScarlet_C1 (Addgene) using the oligonucleotides mScarlet1c [CACCGTTTATGGTGAGCAAGGGCGAGGCAGTG] with mScarlet2c [TGGCGTTTCTACTTGTACAGCTCGTCCATGCCGCC]. For replacement of the tdTomato fluorophore with the mScarlet one, the plasmid pSK536 was amplified with the oligonucleotides pSK536_1 [TGTACAAGTAGAAACGCCATGTCTATCTTCGAGTA] and pSK536_2 [CTCACCATAAACGGTGATGTCTGCTCAAGCGG].

*Af* transformations were performed according to the protocol described in Szewczyk et al. 2006.^69^ Protoplasts were transformed with circular plasmids and transformants were selected by supplementing the media with 0.5 μg/mL pyrithiamine. 10^5^ spore/mL of *Af* transformants were grown in 1 mL of *Aspergillus* Minimal Media (AMM)^68^ at 37°C for 6 h for screening of tdTomato and mScarlet fluorescence using a BD LSR Fortessa system (BD Biosciences) and the BD FACSDIVA 8.0.1 software (Figures S1A and S2A). The results were analyzed using the FlowJo v10 software. The presence and targeted integration of the tdTomato cassette to the *his2At* genomic locus was verified by PRC using tdTomato1 [CAGTTCATGTACGGCTCCAA] with tdTomato2 [AGATGGTCTTGAACTCCACCA] and tdTomato3 [GTAACTACGCTCAACGTGTT] with tdTomato4 [CTTCCTGTTGATGGAATGG] respectively (Figures S1B and S1C). Contrary to tdTomato1 and tdTomato2, which hybridise within the tdTomato sequence, tdTomato3 hybridise to the *ptrA* gene and tdTomato4 to the 3′ *his2A* flanking region, thereby allowing to verify the targeted integration of the tdTomato cassette to the *his2At* genomic locus. Copy number integration of the tdTomato cassette was checked by Southern analysis using a *ptrA*-specific hybridisation probe generated using by PCR with PtrA_SB1 [GGATAGGGGCGAACTTGAACT] and PtrA_SB2 [TTTGGCTGGACTCTCACAAT] (Figures S1B and S1C). The presence of the mScarlet cassette to the *his2At* genomic locus was verified by PCR with mScarlet1 [TCCCCTCAGTTCATGTACGG] and mScarlet 2 [CTTGTACAGCTCGTCCATGC] (Figures S2B and S2C). Targeted integration of a single copy of the mScarlet cassette to the *his2At* genomic locus was checked by Southern blot analysis using *his2A*- and *prtA*-specific hybridisation probes, generated using His2A1 [TTCACCTGATTCAGCTGATTG] with His2A2 [TTCACCTGATTCAGCTGATTG] and PtrA_SB1 [GGATAGGGGCGAACTTGAACT] with PtrA_SB2 [TTTGGCTGGACTCTCACAAT] (Figures S2B and S2C). Genomic DNA for Southern blotting analysis was digested with EcoRI.

#### IFC analysis of *Af*-AEC interaction

The detailed protocol for single-cell analyses of the uptake of tdTomato-expressing *Af* strains by cultured AECs is described in Bertuzzi & Howell, 2021.^39^ For each sample, 2 or 3 acquisitions of 800 (for primary murine AECs) or 8000 single cells (for immortalised AECs and primary human AECs) in focus were performed as technical replicates and each experiment was performed in biological duplicates (for primary AECs) or biological triplicates (for immortalised AECs). *Af* germination on, or within, AECs was enumerated by examination of every AEC_a_ and AEC_i_ using the IDEAS software with biological and technical replicates.

#### FACS-mediated measurements of *Af* viability within AECs

For the preparation of the samples for FACS, the supernatant from cells infected with tdTomato-expressing *Af* strains was collected. Infected cells were washed once with pre-warmed PBS and the wash was pooled together with the supernatants from the infected cells to include floating and loosely attached cells to the analysis. Infected monolayers were dissociated by incubation for 5 min in 1 mL of trypsin-EDTA at 37°C, 5% CO_2_. After 5 min, 1 mL of fresh sDMEM was added and detached cells were collected and pooled together with the supernatant and wash. Cells were centrifuged at 600 g for 3 min. Pellets were resuspended in 500 μL of PBS containing 8 ng/mL CFW to differentially stain AEC_a_ (CFW^+^tdTomato^+^) and AEC_i_ (CFW^−^tdTomato^+^) Samples were incubated in the dark at room temperature for 5 min and then centrifuged at 2400 g for 3 min. Pellets were resuspended in 1 mL of PBS, centrifuged at 2400 g for 3 min and resuspended in 50 μL of PBS for FACS processing using a BD Influx Cell Sorter. Total events were filtered in order to exclude clumps of cells and plotted based on the fluorescent intensity of tdTomato and CFW signal. For each strain and time-point indicated, pools (n = 3–5) of 100 AEC_i_ were sorted in sterile H_2_O. AEC_i_ were lysed by vigorous vortexing and internalised viable *Af* was enumerated following plating of the lysates onto ACM plates and incubation of the plates for 24–48 h at 37°C. The experiment was performed in biological triplicates. As sorting control, for each biological replicate, 5 pools of 100 *Af*^tdT^ spores were sorted in sterile H_2_O and plated in ACM for viable counts. In order to calculate the percentage of killing of intracellular *Af* in AEC_i_ relative to internalised *Af*, data normalisation was performed based on the relative time- and strain-specific stoichiometry coefficient calculated previously.^39^ Statistical analysis was carried out by comparing each time- and strain-specific time point in the analysis with a 100 *Af*^tdT^ spore sorting control.

#### Microfluidic live-cell imaging

mScarlet-expressing *Af* spores were resuspended at a concentration of 10^5^ spore/mL and CFW was diluted to 4 μg/mL suspension in Fluorobrite sDMEM (ThermoFisher). Plates were prepared as from manufacturer’s instructions and 200 μL of FluoroBrite, *Af* spore suspension and 4 μg/mL CFW suspension were added to wells 2, 3 and 5 respectively. *Af* spores were injected onto the A549 monolayers using the Cellasic manifold and the ONIX software (v.5.0) at 37°C, 5% CO_2_. Infection was performed by flowing first fresh FluoroBrite media at 2 psi for 10 min and then alternating pulses of *Af* spores at 4 psi (30 s), 8 psi (30 s) and 0.25 psi (2 min) for 3 cycles. After achieving a satisfactory spore distribution, as visualised using a 20×/0.75NA objective and a Nikon TiS Eclipse microscope, the injection of spores was stopped and plates were incubated for 6 h at 37°C, 5% CO_2_. After incubation, the CFW suspension was injected onto the monolayers at 2 psi for 10 min, followed by a wash with Fluorobrite at 4 psi for 10 min. For the following 14 h of infection, FluoroBrite was set to flow into the chamber at 0.5 psi. These processes were automatically controlled by ONIX software (v.5.0). The 4D multipoint stacked imaging was obtained using a Nikon TiS Eclipse microscope with a 20×/0.75 objective lens, whereby live cell images were taken at 2 h intervals over a duration of 14 h from the injection of CFW. A Hamamtsu Flash 4.0 LT + sCMOS camera with a multiband Semrock filter and Nikon Elements V.4.2 acquisition software was used to capture and collect emitted fluorescence from CFW and mScarlet. Using the Fiji counter,^70^ the number of internalised spores (mScarlet^+^, CFW^−^) and extracellular spores (mScarlet^+^, CFW^+^) was enumerated using the 6 h 3D multipoint stacks and uptake index was calculated as follow:

$$\text{Uptake}\;\text{Index}=\left(\frac{\text{Number}\;\text{of}\;\text{intracellular}\;\text{spores}}{\text{Total}\;\text{number}\;\text{of}\;\text{spores}}\times 100\right)$$

Once identified intracellular *Af* spores, their growth rate was assessed by measuring the length of growing hyphae after 14 h using Fiji.^70^

#### Murine infection and lung processing and staining for flow cytometry

Murine infections were performed under UK Home Office Project Licence PDF8402B7 in dedicated facilities at the University of Manchester. *Af* spores were harvested and prepared as previously described^47^ and viable counts from administered inocula were determined as described previously. Individually vented cages were used to house the mice, which were anesthetized by isoflurane inhalation and infected by intranasal instillation of 40 μL of ATCC46645^tdT^ suspensions (in saline) at the concentration of 2.5 × 10^9^ spores/mL (dosage = 10^8^ spores). Leukopenic mice were immunosuppressed by administration of cyclophosphamide (150 mg/kg, intraperitoneal) on days −3 and −1 and a single subcutaneous dose of hydrocortisone acetate (112.5 mg/kg) administered on day −1. Mice were culled after 4, 8 and 16 h of infection and lungs were lavaged by injection of 2 mL of Dispase solution in HANKS buffer directly into trachea. Whole lungs were collected and tissue digestion was carried out at low speed (100 g) for 1 h at 37°C and lung tissues were minced, filtered and processed in RBC as from the isolation of primary human AECs from lung resections. Following RBC lysis, samples were centrifuged 600 *g* for 6 min at 4°C and the pellets were resuspended in 200 μL of Stop solution. 5 μg/mL of anti-CD16/32 antibody (BioLegend, 101302) were added to block unspecific binding and samples were incubated for 20 min on ice and in the dark. Samples were centrifuged 600 *g* for 6 min at 4°C and the pellets were resuspended in 100 μL of staining mix, containing 1 μg/mL of the antibodies anti-mouse EpCam-PE-Cy7 (118215, Biolegend), an anti-mouse Podoplanin-PE (127407, BioLegend, a marker for type I AECs)^71^ and anti-mouse CD74-FITC (sc-19627, Santa Cruz) in 100 μL of Stop solution. Following incubation for 25 min in the dark and on ice, 1 nM TO-PRO-3 Iodide and 8 ng/mL CFW were added and the samples were incubated for a further 5 min in the dark and on ice. Samples were centrifuged 600 *g* for 6 min at 4°C and the pellets were resuspended in 100 μL PBS for IFC or FACS processing and data analysis as previously specified for *in vitro* experiments. In parallel, single stain-controls were carried out on a processed lung from an uninfected mouse to determine the gating strategy. IFC-acquisition of 800 EpCam^+^ single cells in focus was carried out once for 2 uninfected lungs (not shown), while 4 technical replicates were acquired from 2 pools of 3 lungs obtained from 8-h infected mice (Figure 2B). For FACS (Figure 2D), for each cell type and time-point indicated, 1–5 pools of 100 AEC_i_ from 3 pools of 5 lungs from 4, 8 and 16 h infected mice pools were sorted in sterile H_2_O and internalised viable *Af* was enumerated and analyzed as previously specified.

### Quantification and statistical analysis

GraphPad Prism was used to interpret data and *p* values were calculated through unpaired t tests (with Welch correction as indicated), ordinary 1-way ANOVA with Holm-Sidak’s multiple comparisons test or non-parametric Kruskal-Wallis test with Dunn’s multiple comparisons test, as indicated in each figure legend. Error bars in figures show Standard Deviation (SD). ∗∗∗∗*p* ≤ 0.0001, ∗∗∗*p* ≤ 0.001, ∗∗*p* ≤ 0.01, and ∗*p* ≤ 0.05.
