# Supplementary material for: Epithelial uptake leads to fungal killing in vivo and is aberrant in COPD-derived epithelial cells
Source: iScience. 2024 May 16;27(6):109939. doi: 10.1016/j.isci.2024.109939 (PMC11154633; doi:10.1016/j.isci.2024.109939)
Supplement: Document S1. Figures S1–S3 [file mmc1.pdf]

iScience, Volume *27*

Supplemental information

Epithelial uptake leads to fungal killing *in vivo*

and is aberrant in COPD-derived epithelial cells

Margherita Bertuzzi, Gareth J. Howell, Darren D. Thomson, Rachael Fortune-Grant, Anna Möslinger, Patrick Dancer, Norman Van Rhijn, Natasha Motsi, Alice Codling, and Elaine M. Bignell

Supplemental figure titles and legends

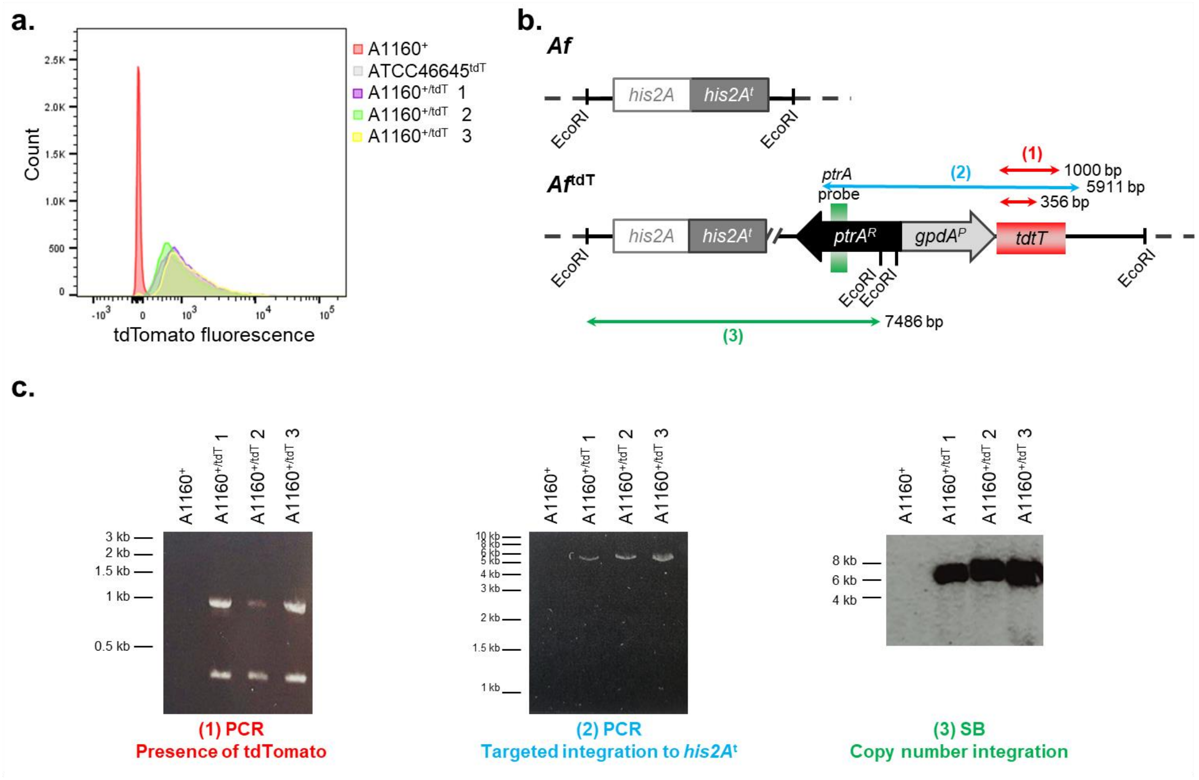

Fig. S1: Design and construction of the tdTomato-expressing *Af* strains (*Af*^{tdT}), used in Figure 1 and Figure 3a-c. (a) Histogram displaying the number of events relative to the tdTomato fluorescence (561 586_15 laser) for the screening of *Af* transformants using flow cytometry. The comparison shows the parental isolate A1160^{+}, the published tdtomato-expressing ATCC46645 isolate[40] and 3 representative A1160$^{+/tdT}$ transformants. **(b)** PCR and Southern blotting strategy for the verification of single, targeted integration of the tdTomato expression construct in A1160^{+}. The presence **(1)** of the tdTomato cassette was verified using the oligonucleotides tdTomato1 and tdTomato2. Two PCR bands are expected from tdTomato-expressing clones (1000 and 356 bp), while no band is expected for the parental isolates A1160^{+}. The targeted integration **(2)** of the tdTomato cassette was verified using the oligonucleotides tdTomato3 and tdTomato4. A single PCR band is expected from tdTomato-expressing clones (5911 bp), while no band is expected for the parental isolates A1160^{+}. Copy number insertion **(3)** of the tdTomato cassette was verified by Southern blotting using

a *prtA*-specific hybridisation probe generated with the oligonucleotides PtrA_SB1 and PtrA_SB2. Genomic DNA for Southern blotting analyses was digested with EcoRI. No band is expected for the parental isolate, whereas a single band of 7486 bp is expected for the reporter strain. **(c)** PCR and Southern blotting for the verification of single, targeted integration of the tdTomato expression construct in representative A1160$^{+/tdT}$ transformants.

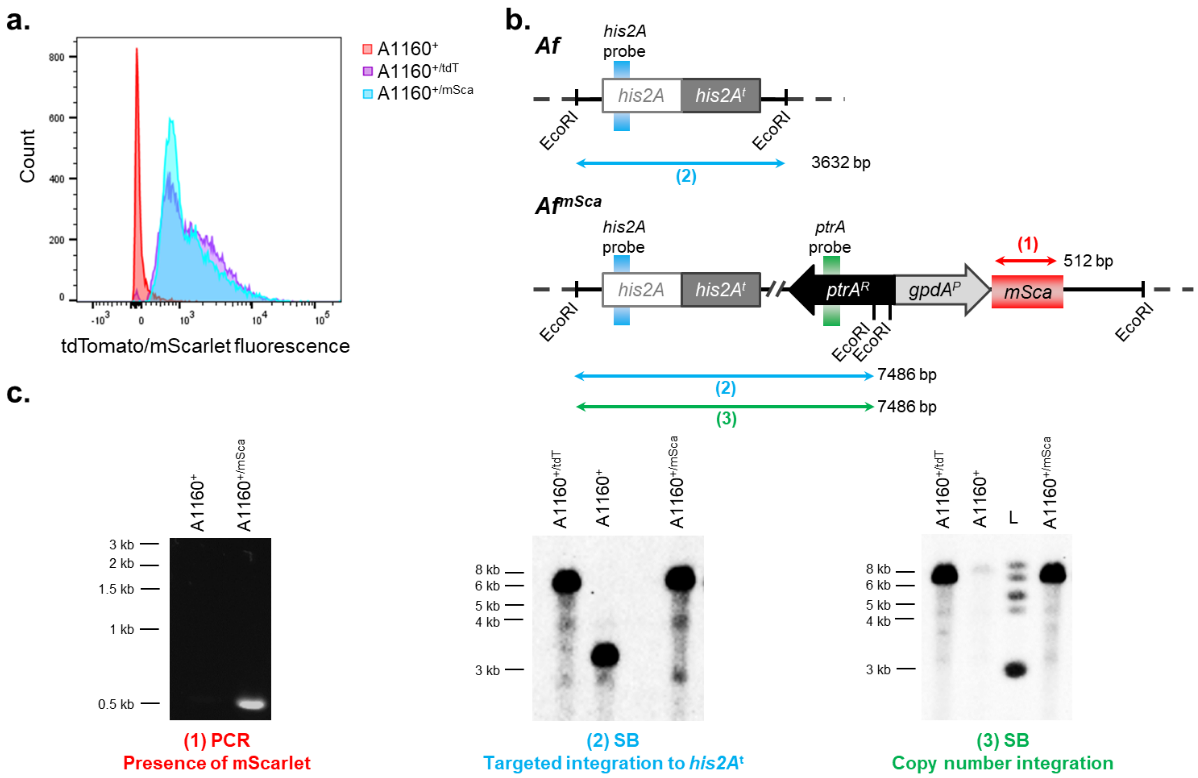

Fig. S2: Design and construction of the mScarlet-expressing *Af* strain (*Af*^{mSca}), used in Figure 3d-e. (a) Histogram displaying the number of events relative to the mScarlet fluorescence (561 586_15 laser) for the screening of *Af* transformants using flow cytometry. The comparison shows the parental isolate A1160^{+}, the representative A1160$^{+/tdT}$ from Fig. S1 and a representative A1160$^{+/mSca}$. **(b)** PCR and Southern blotting strategy for the verification of single, targeted integration of the mScarlet expression construct in A1160^{+}. The presence **(1)** of the mScarlet cassette was verified using the oligonucleotides mScarlet1 and mScarlet2. A PCR band of 512 bp is expected from mScarlet-expressing clones, while no band is expected for the parental isolates A1160^{+}. The targeted **(2)** and single **(3)** integration of the mScarlet cassette was verified by Southern blotting using *his2A*- and *prtA*-specific hybridisation probes, generated using the oligonucleotides His2A1 with His2A2 and PtrA_SB1 with PtrA_SB2. Genomic DNA for Southern blotting analyses was digested with EcoRI. Using the *his2A*-specific hybridisation probe **(2)**, a single band of 3632 bp is expected for parental isolates, while a single band of 7486 bp is expected for the reporter strain. Using the *prtA*-specific hybridisation probe **(3)**, no band is expected for the parental isolate,

whereas a single band of 7486 bp is expected for the reporter strain. **(c)** PCR and Southern blotting for the verification of single, targeted integration of the mScarlet expression construct in A1160$^{+/msca}$. L = protein ladder

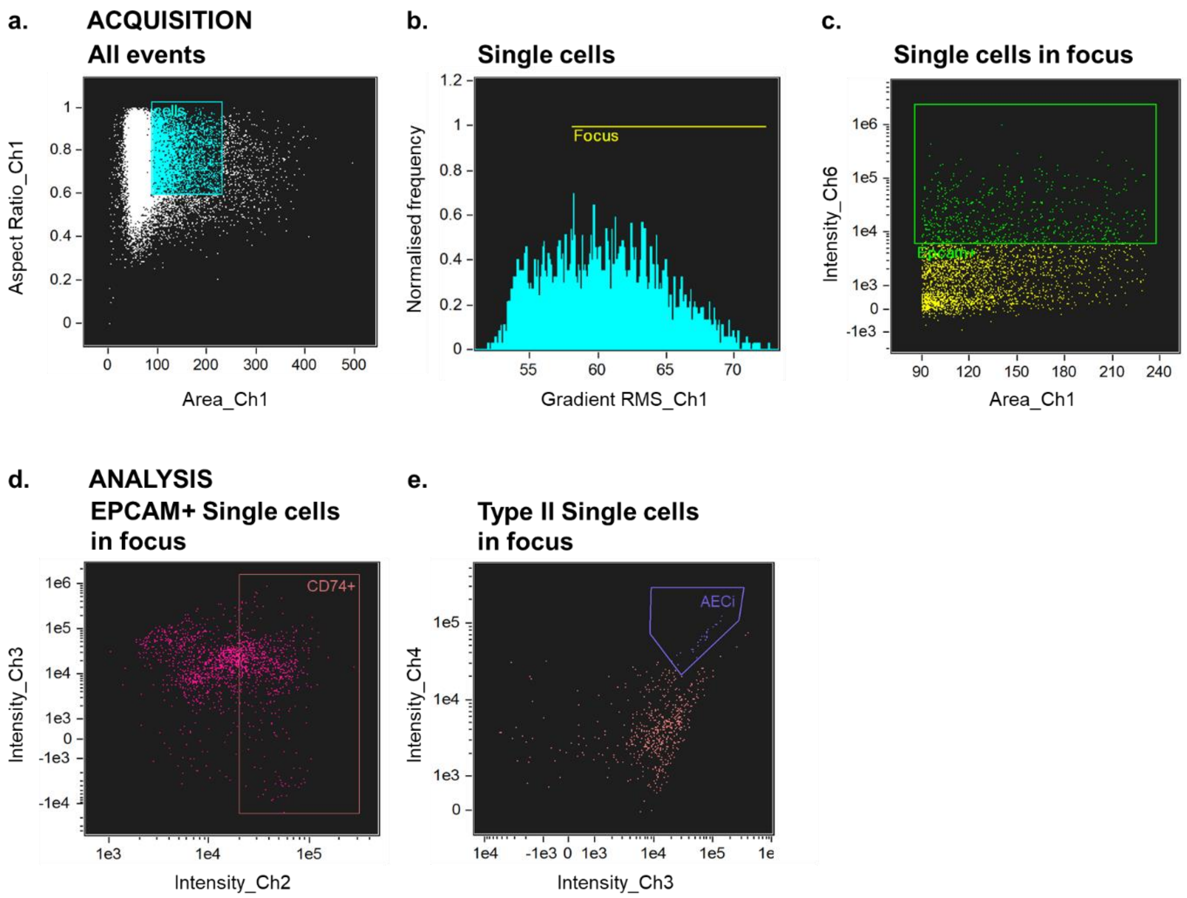

Fig. S3: Representative panels of the gating strategy to determine the percentage of internalising murine type II AECs from dissociated murine lungs infected with *Af*, related to Figure 2. Leukopenic mice were infected with 10^8 spores of ATCC46645^{tdT} for 8 hours. To acquire 2000 EpCAM$^+$ cells, 3 steps are followed: **(a)** single cells are gated from the total events based on Area_Ch1 and Aspect Ratio_Ch1 (which defines the circularity of an object), **(b)** single cells in focus are gated from the single cell based on their normalised frequency relative to the Gradient RMS feature_Ch1 (which define the sharpness of an object) and **(c)** EpCAM$^+$ single cells in focus are gated from the single cells in focus based on their EpCAM fluorescence intensity (Ch_06). Only single cells in focus EPCAM$^+$ cells were acquired for further analysis. To determine the percentage of internalising murine type II AECs (AEC_i), 2 steps are followed: **(d)** type II AECs are gated from 2000 EpCAM$^+$ cells based on their fluorescence intensity on Ch2 (CD74, marker of type II AECs) and Ch3 (Podolanin, marker of type I AECs) and **(e)** AEC_i are gated from type II AECs based on their fluorescence intensity on Ch3 and Ch4 (tdTomato).
